# Age at type 1 diabetes onset does not influence attained brain volume

**DOI:** 10.1186/s12902-025-01868-6

**Published:** 2025-02-18

**Authors:** Tor-björn Claesson, Stefan Mutter, Jukka Putaala, Eero Salli, Daniel Gordin, Per-Henrik Groop, Juha Martola, Lena M. Thorn

**Affiliations:** 1 Department of Radiology, University of Helsinki and Helsinki University Hospital, Helsinki, Finland; 2 Folkhälsan Research Centre, Helsinki, Finland; 3 Research Program for Clinical and Molecular Metabolism, Faculty of Medicine, University of Helsinki, Helsinki, Finland; 4 Department of Nephrology, University of Helsinki and Helsinki University Hospital, Helsinki, Finland; 5 Department of Neurology, University of Helsinki and Helsinki University Hospital, Helsinki, Finland; 6 Minerva Foundation Institute for Medical Research, Helsinki, Finland; 7 Joslin Diabetes Center, Harvard Medical School, Boston, MA USA; 8 Department of Diabetes, Central Clinical School, Monash University, Melbourne, Australia; 9 Baker Heart and Diabetes Institute, Melbourne, VIC Australia; 10 Department of Radiology, Karolinska University Hospital, Stockholm, Sweden; 11 Department of General Practice and Primary Health Care, University of Helsinki and Helsinki University Hospital, PoB 20, Helsinki, FIN-00014 Finland

**Keywords:** Age of onset, Brain, Gray matter, White matter, Magnetic resonance imaging, Diabetes mellitus, type 1

## Abstract

**Introduction:**

Type 1 diabetes is suspected to hamper brain growth, implying that people with earlier diabetes onset would, on average, achieve lower maximal brain volume. We set out to test this hypothesis.

**Methods:**

Examining brain MRI scans of middle-aged people with type 1 diabetes, we related age at diabetes onset to intracranial volume in 180 participants, as well as to cerebral gray and white matter volumes in a subset of 113 (63%) participants, using fractional polynomial regression models. Of the participants, 118 (67%) had been diagnosed with diabetes before 18 years of age.

**Results:**

Of our participants, 54% were women, the median age 40.0 (IQR 33.2–45.0) years and the range of age at diabetes onset was 1.2–39.0 years. We found no association between age at diabetes onset and intracranial volume (*p* = 0.85), cerebral white (*p* = 0.10), or gray matter volumes (*p* = 0.12). Further, correlations between age at diabetes onset and the measured brain volumes were poor in analyses stratified for sex (all correlation coefficients ρ ≤ 0.16).

**Conclusions:**

We found no association between age at diabetes onset and attained intracranial volume or gray or white matter volumes, indicating that type 1 diabetes may not have a clinically significant influence on brain growth.

**Supplementary Information:**

The online version contains supplementary material available at 10.1186/s12902-025-01868-6.

## Introduction

The age at onset of type 1 diabetes has been found to impact cognitive development negatively [1]. This has led to speculation about how much this is caused by organic effects secondary to diabetes and its treatment on the one hand, and social or environmental consequences of diabetes on the other.

Multiple studies have examined associations between age at onset of type 1 diabetes and alterations of brain morphology or function, with differing results [2, 3, 4, 5, 6]. 

Ferguson et al. performed volumetric analysis on brain MRI data, studying total brain volumes and volumes of lateral ventricles, temporal lobes, and amygdalohippocampal complexes. They found larger lateral ventricles in those with onset before seven years of age, than in those with later onset [2]. On the contrary, smaller gray matter volumes have been reported to be more common in those with onset in adolescence, compared to onset at other ages [3]. Furthermore, a recent study on fractional anisotropy and volume fractions (primarily a measure of atrophy) in 69 individuals with type 1 diabetes, aged 20 to 50 years, found no impact of age at diabetes onset, on gray or white matter volume fractions [4]. These studies were limited in sample size, which might affect their ability to detect small effect sizes.

Mazaika et al. reported on a longitudinal study of brain growth in young people with type 1 diabetes, and found differences in growth of both gray and white matter in those with type 1 diabetes compared to controls. Further, the difference in growth rate was larger at lower age [5]. This work was continued by Mauras et al., with the last exams done at 12 years of age, confirming slower growth of gray and white matter in type 1 diabetes, while also demonstrating slower total brain growth [6]. These prospective studies indicate a negative impact of type 1 diabetes on brain volume growth.

In the general population, the growth of gray matter volume is fastest during the first year of life, with volumes peaking at six years of age, while white matter volume growth is slower, peaking around 30 years of age [7]. 

If type 1 diabetes impairs brain growth during childhood, then earlier disease onset would mean longer exposure to this growth impairment. We therefore hypothesized that earlier disease onset would be related to a more pronounced negative effect on maximally attained brain volume. Our aim was, therefore, to study the association between age at diabetes onset and intracranial volume in a well-characterized cohort of adult individuals with type 1 diabetes.

## Methods

### Study population

Participants were recruited from the nationwide Finnish Diabetic Nephropathy (FinnDiane) Study, ongoing since 1997 with the purpose of identifying risk factors for micro- and macrovascular complications of type 1 diabetes [8]. Enrollment in this brain MRI sub-study took place between 2011 and 2017. We were able to enlist 191 FinnDiane Study participants aged 18 to 50 years. Exclusion criteria before MRI imaging were contraindications for MRI, kidney replacement therapy, or any pre-existing cerebrovascular disease, as verified by the validated Questionnaire for Verifying Stroke-Free Status [9]. 

Type 1 diabetes was defined as disease onset before 40 years of age and start of insulin treatment within one year of diagnosis.

For this study, we excluded participants with brain parenchymal changes suspected of influencing gray or white matter volume segmentation (the process of labeling voxels during volumetric analysis). For the intracranial volume analysis, we excluded 11 participants: 2 with arachnoid cysts, 3 with widespread cerebral calcification, 1 with previous brain surgery, 1 with a dural fistula, 1 with focal cortical dysplasia, and 3 with developmental venous anomalies, leaving 180 participants for analysis. For analyses of gray and white matter, we excluded an additional 67 participants: all participants with MRI signs of cerebral small vessel disease (out of concern that white matter hyper-intensities might affect white matter segmentation, since a voxel has exactly one label), 2 participants with MRI signs of demyelinating disease, and 1 with temporal contusions, leaving 113 participants for analysis.

### Clinical examination

All participants were subjected to a clinical examination, including anthropometry, blood pressure measurements, and blood analyses including HbA_1c_, creatinine, and lipids. A thorough medical history was taken, including current medication, smoking, and manifestations of cardiovascular disease. The age at diabetes onset was calculated as the time from birth date to diabetes onset and expressed in years. The FinnDiane Study only registers diabetes onset year, so for the purpose of this study, we used data on diabetes diagnosis from two registries (Reimbursement Entitlements of Drug Expenses, Kela and the Care Register for Health Care, Finnish Institute for Health and Welfare) to obtain the exact date when a diabetes diagnosis first appeared in public health records.

### Brain MRI

Brain MRI examinations were performed at the Helsinki Medical Imaging Center of the Helsinki University Hospital within one year of the clinical examination. We used a single 3T scanner (Achieva, Philips, Best, The Netherlands) with standardized acquisition protocols. Acquisition parameters are given in Supplemental Table 1. All scans were reviewed by an experienced neuroradiologist (J.M.), as has been described previously [10]. 

To characterize the impact of age at diabetes onset on brain volumes, our primary outcome variable was total intracranial volume, serving as a surrogate for maximally attained brain volume. This obviates the need to compensate for age, under the usual assumption that intracranial volume does not shrink after being established as sutures close at the end of brain maturation [11]. We further examined total supra-tentorial gray and white matter volumes, here including age to compensate for atrophy.

### Volumetric analysis

We analyzed the MPRAGE image stacks using the standard cross-sectional analysis pipeline of FreeSurfer 6.0 (http://surfer.nmr.mgh.harvard.edu/) [12]. The analyzes were corrected and rerun until the segmented pial surface and gray-white matter boundary conformed to the image data, as visualized on a clinical grade workstation and environment at standard window settings by a radiologist (T.C.). We converted all voxel values to mm³ (https://surfer.nmr.mgh.harvard.edu/fswiki/BrainVolStatsFixed). The FreeSurfer variable Etiv, calculated using atlas normalization, was used for intracranial volume [11]. Gray matter volume was calculated as the sum of FreeSurfer variables CortexVol and bilateral Thalamus-Proper, Amygdala, Caudate, Putamen, Pallidum, and Hippocampus. White matter volume was read from the CerebralWhiteMatterVol variable.

### Statistical analysis

Correlation coefficients were calculated using Spearman’s method.

In order to retain study power, we examined the impact of age at disease onset using regression analysis, rather than by dichotomizing participants based on age at onset cut points [13]. 

Since some previous studies have indicated a larger impact of both an earlier disease onset, as well as of one in adolescence, we suspected that the relationship between age at onset and intracranial volumes might be bimodal. For this reason, we chose to evaluate these relationships using fractional polynomial models, which are extended generalized linear models, with associated methods for model and covariate selection (allowing curve shapes other than the linear first grade *y = ax + b).*

For the models, covariates were eliminated in an iterative fashion using a procedure closely related to backfitting [14]. In addition to age at diabetes onset, all initial regression models included HbA_1c_, sex, age, and height as predictors. Sex was included since brain size differs between sexes [15]. Height was included since type 1 diabetes has historically been shown to affect adult height (although it is uncertain how relevant this is with modern insulin therapy) [16], and a weak correlation has been reported between height and some of the cerebral volumes under study [17]. 

We permitted a maximum of four degrees of freedom, that is a maximum permitted degree m = 2, for fractional polynomial transformation, and always retained the age at onset-factor during model selection.

To further try to find any association between age at disease onset, we repeated all analyses post hoc with participants stratified by sex. As a quality control, we also redid the analyses without those participants who developed diabetes at ≥ 18 or ≥ 30 years of age (when gray and white matter volume, respectively, have peaked).

R version 4.3.2 was used for statistical analyses [18], with statistical significance set at *p* < 0.05. The tidyverse package was used for data processing [19], and the mfp package was used for fractional polynomial regression (https://cran.r-project.org/web/packages/mfp/). Graphics were produced using ggplot2 and tables using gtsummary [20, 21]. 

## Results

Clinical characteristics of the study participants, grouped according to the overlapping cohorts selected for the respective models, are presented in Table 1. The sex distribution was similar in both models.

**Table 1:** Clinical characteristics of the study cohort per regression model

Characteristic	Intracranial volume analysis cohort	Gray and white matter volume analysis cohort
N	180	113
Women, N (%)	97 (54%)	62 (55%)
Age, years	40.0 (33.2, 45.0)	37.4 (32.7, 41.9)
Age at onset, years	13.4 (7.5, 21.7)	13.9 (6.7, 21.6)
Diabetes duration, years	21.8 (18.3, 31.3)	21.6 (18.3, 28.2)
Height, cm	173 (167, 180)	173 (166, 180)
Body mass index, kg/m²	26.4 (23.8, 29.3)	26.1 (23.8, 29.8)
Systolic blood pressure, mmHg	129 (119, 138)	126 (117, 136)
Diastolic blood pressure, mmHg	76 (71, 82)	75 (70, 82)
HbA1c, %	8.10 (7.40, 8.80)	8.00 (7.30, 8.70)
HbA1c, mmol/mol	65 (57, 72)	64 (56, 71)
Creatinine, mmol/L	69 (61, 79)	68 (60, 79)
Total cholesterol, mmol/L	4.40 (4.00, 4.91)	4.42 (4.07, 4.99)
LDL cholesterol, mmol/L	2.37 (2.00, 2.87)	2.47 (2.05, 2.99)
HDL cholesterol, mmol/L	1.51 (1.25, 1.80)	1.48 (1.25, 1.78)
Triglycerides, mmol/L	0.89 (0.69, 1.27)	0.89 (0.66, 1.30)
Antihypertensive medication, N (%)	66 (37%)	34 (30%)
Lipid-lowering medication, N (%)	38 (21%)	19 (17%)
Retinal photocoagulation, N (%)	43 (24%)	23 (20%)
Coronary heart disease, N (%)	1 (0.6%)	1 (0.9%)
Current smoking, N (%)	14 (7.8%)	11 (9.7%)
Total intracranial volume, mL	1,603 (1,486, 1,739)	1,578 (1,473, 1,712)
White matter volume, mL	465 (421, 505)	454 (411, 496)
Total gray matter volume, mL	656 (622, 706)	655 (622, 704)

*Continuous variables given as median (IQR)*,* categorical variables as N (%).*

**Fig. 1:**
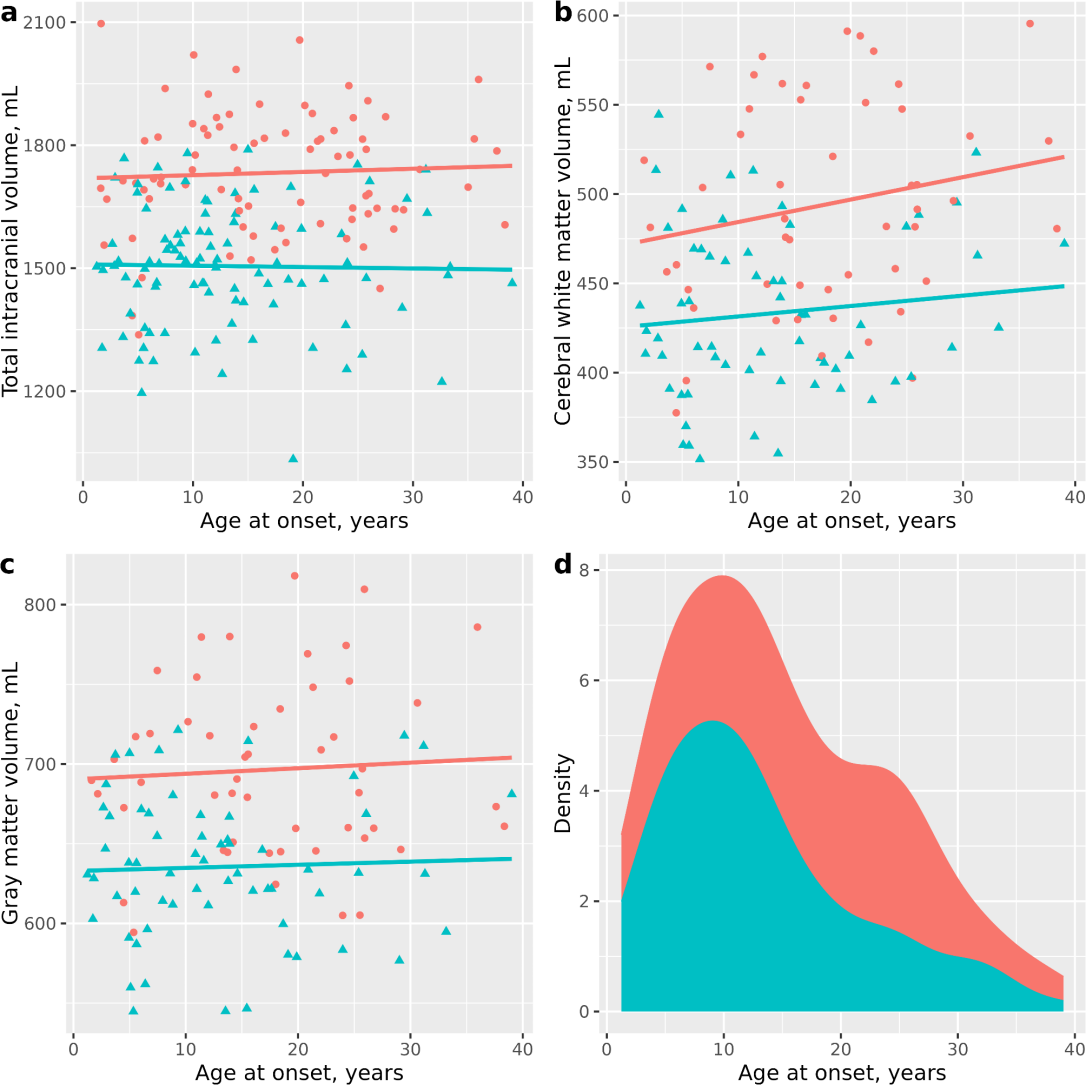
The relationship between age at diabetes onset and brain volumes. The relationship between age at type 1 diabetes onset and intracranial (**a**), White matter (**b**) and gray matter (**c**) volumes, as well as a density plot of Age at diabetes onset by sex (**d**). Panels a and d show data for all participants, while b–c only display data for those included in the specific regression model. Women in blue, men in red

The relationships between age at disease onset and the studied volumes are visualized in Fig. 1a–c, where the spread of volume vs. age at onset is plotted per sex, together with a first-degree ordinary least squares regression line. Most regression lines had a slightly positive slope, but the spread of volumes vs. age at disease onset was wide. Correlations were subsequently weak, with correlation coefficients ρ = 0.255, *p = 0.26* (men) and ρ = 0.052, *p = 0.62* (women) for cerebral white matter volume vs. age at onset, ρ = 0.006, *p = 0.96* (men) and ρ = -0.006, *p = 0.96* (women) for intracranial volume vs. age at onset, and ρ = 0.007, *p = 0.96* (men) and ρ = -0.016, *p = 0.90* (women) for gray matter volume. Figure 1d shows the distributions of age at diabetes onset by sex.

The associations between age at type 1 diabetes onset and intracranial, gray, and white matter volumes are presented in Table 2. Age at diabetes onset was not associated with the studied volumes in any of the models.

**Table 2:** Association between age at diabetes onset and intracranial, white matter, and gray matter volumes

	Intracranial volume, *N* = 180			White matter volume, *N* = 113			Gray matter volume, *N* = 113		
Characteristic	Beta	95% CI	*p*-value	Beta	95% CI	*p*-value	Beta	95% CI	*p*-value
Age at diabetes onset, years	0.231	-2.24, 2.70	0.854	0.888	-0.178, 1.96	0.102	0.771	-0.213, 1.75	0.123
Sex, women	-227	-272, -182	< 0.001	-56.6	-76.5, -36.7	< 0.001	-36.8	-61.2, -12.5	0.003
Age, years							-2.75	-4.05, -1.46	< 0.001
Height, cm							2.09	0.839, 3.34	0.001

*Multivariable models. Age and height were only retained in the gray matter model. All volumes in mL. CI = Confidence Interval*

Excluding participants with age at onset before either 18 or 30 years of age did not cause significant change in the results of modeling (Supplemental Tables 2 and 3), nor did performing the analyses with participants split by sex (Supplemental Tables 4 and 5).

## Discussion

We studied the impact of age at diabetes onset on total intracranial, gray matter, and white matter volumes in a well-characterized cohort of adults with type 1 diabetes. Correcting for sex, we found no associations between age at type 1 diabetes onset and the studied volumes, indicating that attained brain size in type 1 diabetes is not affected by the age at diabetes onset in this cohort. The results, thus, do not support our research hypothesis.

This negative finding between age at type 1 diabetes onset and brain volumes in adults can be a result of multiple factors. It can be either a true negative finding or represent a type II error. Our intracranial volume model included 180 participants. However, many of those had a diabetes onset in adult age. Excluding those with later diabetes onset from the analysis (Supplemental Tables 2 and 3) did not change the results, but the sample size is even smaller in those models. Our analysis of gray and white matter volumes, on the other hand, suffers from a small number of participants (113), and is further hampered by the lack of normalization, which is typically used to correct for individual differences in brain size [15]. Also, these volumes could be affected by a myriad of other factors, including normal volume loss related to aging [7]. We tried to compensate for this by splitting the participants by sex (Supplemental Tables 4 and 5), resulting in even smaller sample sizes. For these reasons, our negative result in these brain volume analyses should not be interpreted as strong evidence against age at diabetes onset causing impaired brain growth. However, our results are clear, in that the association and correlation between age at diabetes onset and intracranial volume in our cohort is poor. This, together with the low R^2^, (0.001–0.040) indicates that, even if this was a type 2 error, the missed effect would be small.

Two longitudinal studies have examined brain growth in children with type 1 diabetes. One followed 111 children with type 1 diabetes and 50 controls, finding no brain growth differences [22]. This result agrees with our finding of no association between age at diabetes onset and intracranial volume. The second, by Mauras et al., [6] followed 144 children with type 1 diabetes and 50 controls. In contrast to our study, they found smaller brain volumes in the type 1 diabetes group compared to controls. Further, the size difference increased at follow-up, indicating impaired growth. The difference was small, around 3% at 12 years of age, and it is possible that our sample size of 180 is insufficient for detecting the impact this growth difference would have on intracranial volume. Also, we were looking at an indirect measure of brain growth, while Mauras et al. followed brain growth as it happened, which allows for more accurate measurements.

As for brain volumes, a cross-sectional study of middle-aged people with type 1 diabetes has examined gray and white matter volume fractions (that is total gray and white matter normalized to total intracranial volume) in middle-aged people with type 1 diabetes did not find an impact of age at diabetes onset [4]. This study aimed to capture atrophy and not volumes of gray and white matter per se. Volume fractions are commonly used when examining brain atrophy. Volume fractions are, however, problematic in studies of reduced brain growth, since reduced brain growth reasonably would result in a smaller intracranial volume, reducing both dividend (the studied brain volume) and divisor (intracranial volume). Effects on brain growth are therefore unlikely to cause changes in volume fractions, and we consider this an inappropriate measure for studying effects on brain growth.

The age at onset distribution in our cohort shows a peak at diabetes onset of 10 years of age in both sexes, but men show another peak at approximately 25 years. We do not have a clear explanation for this second peak, but one potential explanation could be recruitment bias. The FinnDiane Study only recruits adults with type 1 diabetes, and young adults with new onset type 1 diabetes could be both interested in participating and targeted by recruitment nurses. In type 1 diabetes brain MRI studies, the age at type 1 diabetes onset tends to vary depending on study design, with cohorts including only children typically having a lower age at onset [22], while the Diabetes Control and Complications Trial/Epidemiology of Diabetes Interventions and Complications (DCCT/EDIC) Study report a mean of 22 years [23].

It is unfortunate that we do not have access to childhood HbA_1c_ data for our participants, since it would have let us evaluate the effect of long-standing childhood hyperglycemia in our material.

Given the poor correlation between brain size and cognitive function [24], the significance of small changes, such as those reported by Mauras et al. [6] is uncertain, and the possibility that the association between diabetes onset and cognitive function [1] could be mediated by factors more subtle than smaller brains should be considered. Type 1 diabetes has been shown to be associated with accelerated brain atrophy in later life in the DCCT/EDIC Study, with higher HbA_1c_ linked to smaller gray matter volume [23]. If the mechanism behind this accelerated atrophy is active also during brain development, the gray and white matter volumes in those with earlier disease onset could be more affected (as opposed to intracranial volume), but we could not observe indications of this in our cohort.

Limitations of the present study are its cross-sectional nature, not having measured blood glucose at the time of MRI (this is known to dynamically affect brain volumes, and it might have been beneficial to correct for blood glucose levels at time of MRI), or having access to data on childhood glycemic control. A major strength is the thorough review of our MRI material, and the fact that we avoid undue dichotomization based on age at diabetes onset [13]. 

Taken together, we found no association between age at diabetes onset and intracranial volume, or brain volumes, in adulthood in patients with type 1 diabetes in contrast to our study hypothesis. The good news is that, if there is an impact of age at diabetes onset on brain growth, this impact would be small in magnitude and probably not clinically relevant.

## Electronic supplementary material

Below is the link to the electronic supplementary material.

Supplementary Material 1

## Data Availability

Individual-level data for the study participants are not publicly available because of restrictions guaranteed to the participant at the time of data collection. The Readers may propose collaboration to research the individual level data with correspondence with the lead investigator.
