# Supplementary material for: Age at type 1 diabetes onset does not influence attained brain volume
Source: BMC Endocr Disord. 2025 Feb 18;25:43. doi: 10.1186/s12902-025-01868-6 (PMC11834590; doi:10.1186/s12902-025-01868-6)
Supplement: Supplementary file 1 — Supplementary Material 1 [file 12902_2025_1868_MOESM1_ESM.docx]

Online materials

*Supplemental table 1: MRI sequence parameters*

| **Sequence** | **TR** | **TE** | **Flip angle** | **Echos** | **Voxel size** |
| --- | --- | --- | --- | --- | --- |
| T1 MPRAGE | shortest | 4.6 ms | 8° | 1 | 0.88x0.88x0.88 mm |
| 3D TOF | 25 ms | 3.5 ms | 20° | 1 | 0.28x0.54x0.50 mm |
| 3D SSH | shortest | 46 ms | 90° | 1 | 2.00x2.00 mm |
| T1 IR TSE | 2,000 ms | 10 ms | (refocus 120°) | 1 | 0.98x1.22 mm |
| T2 FFE | shortest | 16 ms | 18 | 1 | 0.90x1.12 mm |
| T2 FLAIR | 11,000 ms | 125 ms | (refocus 120°) | 1 | 0.65x0.87 mm |
| T2 TSE | 4,000 ms | 80 ms | 90° | 1 | 0.60x0.75 mm |
| VEN BOLD | shortest | shortest | 10° | 1 | 1.00x0.99x0.50 mm |

| Supplemental table 2: Multivariable model, including only participants with age at diabetes onset < 18 years. All volumes in mL. | | | | | | | | | |
| --- | --- | --- | --- | --- | --- | --- | --- | --- | --- |
| **Characteristic** | **Intracranial volume, N=118** | | | **White matter volume, N=73** | | | **Gray matter volume, N=73** | | |
|  | **Beta** | **95% CI1** | **p-value** | **Beta** | **95% CI1** | **p-value** | **Beta** | **95% CI1** | **p-value** |
| Age at diabetes onset, years | 03.14 | -2.93, 9.21 | 05.08 | 08.00 | -2.06, 3.02 | 11.48 | 01.08 | -1.99, 2.13 | 15.48 |
| Sex, women | −214 | -269, -159 | <0.001 | -54.4 | -79.9, -28.9 | <0.001 | -42.8 | -70.5, -15.1 | 00.03 |
| Height, cm |  |  |  |  |  |  | 01.56 | 0.073, 3.04 | 00.40 |
| Age, years |  |  |  |  |  |  | −02.31.00 | -3.74, -0.887 | 00.02 |

| Supplemental table 3: Multivariable model, including only participants with age at diabetes onset < 30 years. All volumes in mL. | | | | | | | | | |
| --- | --- | --- | --- | --- | --- | --- | --- | --- | --- |
| **Characteristic** | **Intracranial volume, N=168** | | | **White matter volume, N=105** | | | **Gray matter volume, N=105** | | |
|  | **Beta** | **95% CI1** | **p-value** | **Beta** | **95% CI1** | **p-value** | **Beta** | **95% CI1** | **p-value** |
| Age at diabetes onset, years | −00.36.00 | -3.11, 3.04 | 16.22 | 05.01 | -1.04, 1.65 | 10.58 | 05.29 | -0.885, 1.54 | 09.52 |
| Sex, women | −225 | -272, -178 | <0.001 | -58.5 | -79.5, -37.5 | <0.001 | -38.0 | -62.7, -13.2 | 00.03 |
| Age, years |  |  |  |  |  |  | −03.12.00 | -4.03, -1.41 | <0.001 |
| Height, cm |  |  |  |  |  |  | 02.08 | 0.820, 3.34 | 00.01 |

| Supplemental table 4: Multivariable model, including only men. Five outliers were excluded from the white matter model and six from the gray matter model. All volumes in mL. | | | | | | | | | |
| --- | --- | --- | --- | --- | --- | --- | --- | --- | --- |
| **Characteristic** | **Intracranial volume, N=83** | | | **White matter volume, N=46** | | | **Gray matter volume, N=45** | | |
|  | **Beta** | **95% CI1** | **p-value** | **Beta** | **95% CI1** | **p-value** | **Beta** | **95% CI1** | **p-value** |
| Age at diabetes onset, years | 12.58 | -2.72, 4.27 | 10.59 | 01.38 | -0.432, 3.19 | 02.12 | 07.29 | -1.12, 2.02 | 09.26 |
| Height, cm |  |  |  |  |  |  | 03.36 | 1.25, 05.07 | 00.01 |
| Age, years |  |  |  |  |  |  | −03.51.00 | -5.79, -1.22 | 00.03 |

| Supplemental table 5: Multivariable model, including only women. All volumes in mL. | | | | | | | | | |
| --- | --- | --- | --- | --- | --- | --- | --- | --- | --- |
| **Characteristic** | **Intracranial volume, N=97** | | | **White matter volume, N=62** | | | **Gray matter volume, N=62** | | |
|  | **Beta** | **95% CI1** | **p-value** | **Beta** | **95% CI1** | **p-value** | **Beta** | **95% CI1** | **p-value** |
| Age at diabetes onset, years | 12.58 | -2.72, 4.27 | 10.59 | 01.38 | -0.432, 3.19 | 02.12 | 07.29 | -1.12, 2.02 | 09.26 |
| Height, cm |  |  |  |  |  |  | 03.36 | 1.25, 05.07 | 00.01 |
| Age, years |  |  |  |  |  |  | −03.51.00 | -5.79, -1.22 | 00.03 |
